# Efficient talent identification in women’s football: A ranking-based approach for goal scoring analysis

**DOI:** 10.1371/journal.pone.0342115

**Published:** 2026-02-24

**Authors:** Songyi Song, Hee-Su Kim

**Affiliations:** 1 Sports Information Science Laboratory, Dankook University, Yongin-si, Gyeonggi-do, Republic of Korea; 2 College of Business, Hankuk University of Foreign Studies, Seoul, Republic of Korea; Portugal Football School, Portuguese Football Federation, PORTUGAL

## Abstract

Individual goal-scoring analysis in women’s football faces severe class imbalance and limited scouting resources, where classification metrics alone do not capture operational efficiency. We analyzed 2,535 non-goalkeeper player-match observations from the 2023 FIFA Women’s World Cup (736 unique players) with 51 performance features, excluding match-outcome variables to emphasize individual actions. Using nested cross-validation, LightGBM captured 79.4% of goal-scoring observations within the top 20% of ranked observations; an out-of-bag (OOB) bootstrap gains analysis yielded 73.9% capture at Top 20% (lift = 3.69x; 95% CI: 63.9%−84.3%). Permutation and SHAP consensus highlighted tactical availability (Total Offers) and combined technical/physical workload indicators (Passes Attempted, Jogging Distance, Top Speed). This proof-of-concept study shows that ranking-based evaluation improves scouting efficiency using basic match statistics, while thresholds and feature weights require validation in other competitive contexts.

## 1. Introduction

Women’s football has grown rapidly, yet analytical infrastructure and access to tracking data often lag behind the men’s game, leaving many organizations reliant on basic match statistics [1]. This gap motivates cost-effective methods tailored to women’s football characteristics and technical-tactical differences from the men’s game [2–4].

Goal scoring is a rare outcome in elite tournaments, creating severe class imbalance and a large pool of player-match observations to review. For talent identification, the operational question is not only accuracy but how efficiently limited scouting resources can surface likely scorers.

Prior football ML studies have largely focused on team-level outcomes or emphasized classification metrics [5–8]. Work on women’s tournament data shows the feasibility of machine learning for goal and shot prediction [9], but most studies still evaluate performance with threshold-based metrics that do not directly quantify scouting workload or coverage [10,11].

Recent women’s football analytics also span interpretable xG models, goal-event behavioral analysis, and cross-gender tactical comparisons, while spatial movement models provide broader methodological context for football analytics [12–15].

Ranking-based evaluation addresses this gap by measuring how many goal-scoring observations are captured within the top-ranked fraction of candidates (e.g., gains/lift charts) [16–18]. To support reliable inference in a limited tournament setting, we pair this evaluation with nested cross-validation and bootstrap uncertainty estimation, and we validate feature importance using complementary permutation and SHAP methods [19–21].

This study addresses the gap between evaluation metrics and operational utility in women’s football analytics with three questions:

1. **Efficiency:** Do ranking-based evaluations improve goal-scorer identification efficiency over traditional approaches?
2. **Features:** Which patterns emerge from permutation + SHAP consensus, and how do they inform talent identification?
3. **Stability:** Which algorithm provides the most reliable efficiency estimates under **nested cross-validation**?

Key contributions:

- **Methodological Innovation:** Gains Chart evaluation with **nested cross-validation** and bootstrap CIs.
- **Practical Framework:** Scalable approach using basic match statistics accessible to resource-constrained organizations.
- **Feature Insights:** Permutation + SHAP consensus with leakage-free features to isolate primarily individual-focused talent signals.

We analyze the 2023 FIFA Women’s World Cup to provide a reproducible, resource-efficient scouting framework for women’s football.

## 2. Materials and methods

### 2.1. Data source and preprocessing

#### 2.1.1. Dataset description and collection.

Data for this study were obtained from the 2023 FIFA Women’s World Cup, encompassing all 64 matches played between July 20 and August 20, 2023. The tournament featured 32 national teams, providing a comprehensive dataset of elite women’s football performance at the highest international level.

Our analysis focused on 2,535 non-goalkeeper field player-match observations from athletes who participated in at least one match during the tournament (736 unique players across the full dataset). Goalkeepers were excluded due to fundamentally different performance profiles and goal-scoring expectations. The dataset comprised 51 performance features collected through official FIFA match statistics and categorized into four primary domains:

**Physical Performance Metrics (n = 18):** Distance covered (total, high-intensity, sprint), speed measurements (maximum, average), acceleration and deceleration events, time spent in different intensity zones.

**Technical Performance Indicators (n = 22):** Passing statistics (completed, accuracy, progressive), shooting metrics (attempts, on target), ball control measures (touches, successful dribbles), defensive actions (tackles, interceptions, clearances).

**Tactical Positioning Data (n = 10):** Average field positions, positioning patterns, time spent in different field thirds, spatial movement characteristics.

**Methodological Clarification:** This is a retrospective analysis using post-match data to identify indicators for future scouting, not real-time match prediction.

#### 2.1.2. Target variable definition and class distribution.

The target variable indicated whether a player scored >= 1 goal in a given player-match observation, creating a binary classification problem with significant class imbalance:

- **Goal-scoring observations (Positive Class):** 136 observations (5.4%)
- **Non-goal-scoring observations (Negative Class):** 2,399 observations (94.6%)
- **Class Imbalance Ratio:** 17.6:1

This extreme imbalance reflects the reality of elite football competition, where goal-scoring opportunities are limited and distributed among a small subset of players.

#### 2.1.3. Data quality assessment and preprocessing.

**Exclusion of Match-Outcome Variables:** To minimize post-hoc leakage and emphasize individual player actions, we excluded match-outcome-related variables such as TeamScore, Win/Loss, and Game Number. Pre-event team ranking (June 2023, prior to tournament) was retained as a contextual control.

**Missing Value Analysis:** Initial data quality assessment revealed missing values in 8.3% of observations, primarily due to players with limited match time. Missing value patterns were analyzed to confirm random distribution.

**Imputation Strategy:**

- Continuous variables: Median imputation within position groups
- Remaining missing values: Overall median imputation after position-level fill
- Preservation of data distribution characteristics

**Outlier Detection and Treatment:** Numeric features were winsorized to the 5th and 95th percentiles after imputation to limit the influence of extreme values while preserving distribution shape.

**Feature Standardization:** Standardization (z-score) was applied only within the Logistic Regression and SVM pipelines via StandardScaler inside the cross-validation loop. Tree-based models were trained on unscaled features.

### 2.2. Machine learning algorithm implementation

#### 2.2.1. Algorithm selection and rationale.

Five machine learning algorithms were selected to represent different methodological approaches and assess ranking consistency across diverse models:

**Tree-Based Ensemble Methods:**

- **LightGBM:** Efficient gradient boosting implementation with fast training [22].
- **Random Forest:** Bagging with random feature selection for robust predictions.
- **XGBoost:** Gradient boosting baseline [23].

**Linear and Non-linear Methods:**

- **Logistic Regression:** Linear baseline with L2 regularization for interpretability.
- **Support Vector Machine:** Non-linear classification with RBF kernel for high-dimensional data.

Each algorithm was selected based on proven effectiveness in sports analytics applications and ability to generate probabilistic rankings for Gains Chart evaluation.

#### 2.2.2. Nested cross-validation framework.

To ensure reproducibility and generalizability, we implemented **nested cross-validation** with the following structure:

**Outer Loop (Model Evaluation):**

- 5-fold stratified cross-validation
- Maintains class distribution (17.6:1 ratio) in each fold
- Provides unbiased performance estimates

**Inner Loop (Hyperparameter Optimization):**

- 3-fold stratified cross-validation within each outer training set
- Grid search across algorithm-specific parameter spaces
- F1-score optimization for hyperparameter selection

**Two-Stage Metric Selection:** Hyperparameters were tuned by F1-score in the inner loop to ensure balanced classification performance; final model selection used Capture Rate @ Top 20% to align with scouting efficiency.

**LightGBM Parameters:**

- n_estimators: [100, 200]
- max_depth: [16,24]
- learning_rate: [0.05, 0.1]
- class_weight: ‘balanced’

**XGBoost Parameters:**

- n_estimators: [100, 200]
- max_depth: [16,24]
- learning_rate: [0.05, 0.1]
- scale_pos_weight: automatically calculated as (n_negative/ n_positive) to handle class imbalance

**Random Forest Parameters:**

- n_estimators: [100, 200]
- max_depth: [1,16]
- min_samples_split: [6,16]
- class_weight: ‘balanced’

**Logistic Regression Parameters:**

- C: [0.1, 1, 10]
- class_weight: ‘balanced’

**SVM Parameters:**

- C: [0.1, 1, 10]
- kernel: ‘rbf’
- gamma: ‘scale’
- class_weight: ‘balanced’

This nested structure prevents information leakage between hyperparameter tuning and model evaluation, improving the robustness and reproducibility of performance estimates.

**Class Imbalance Handling:** Class imbalance (17.6:1 ratio) was handled using cost-sensitive weighting (class_weight = ‘balanced’) rather than synthetic oversampling (e.g., SMOTE, ADASYN [25,26]) to preserve the original data distribution and ranking geometry.

#### 2.2.3. Model selection criteria.

To ensure transparent and reproducible model selection, we pre-specified the following selection criterion before conducting the analysis:

**Primary Selection Criterion:** Capture Rate @ Top 20% (ranking efficiency)

As motivated in the Introduction, model selection prioritized ranking efficiency. All five candidate algorithms were evaluated under identical nested CV conditions, and the algorithm with the highest Capture Rate @ Top 20% was selected as the primary model (results in Section 3.1). This pre-specification follows best practices for avoiding selection bias in machine learning studies [27].

#### 2.2.4. Permutation test for significance.

To assess whether the observed ranking performance exceeds chance, we conducted a target shuffling (Y-randomization) permutation test. The goal-scoring labels were randomly permuted 100 times while preserving the feature matrix. For each permutation, we trained LightGBM using the best hyperparameters identified from the full hyperparameter search and computed Capture Rate @ Top 20% using 5-fold cross-validation. This produced a null distribution of ranking performance under no signal. The observed capture rate was 0.764, exceeding the null mean of 0.198, yielding p = 0.0099.

#### 2.2.5. Bias mitigation strategies.

Potential biases were mitigated through design choices embedded throughout the Methods. Selection bias was addressed by the nested cross-validation protocol that separates hyperparameter tuning from performance estimation (Section 2.2.2) [27]. Team-dependency and post-hoc leakage were reduced by excluding match-outcome variables during preprocessing (Section 2.1.3). Overfitting and spurious signal were assessed via Y-randomization permutation testing (Section 2.2.4). To reduce analytic flexibility, the primary operational metric (Capture Rate @ Top 20%) was pre-specified for final model selection and emphasized in reporting (Sections 2.2.3 and 2.4). Finally, feature-importance was triangulated across complementary methods (Section 2.3).

### 2.3. Multi-method feature importance analysis

#### 2.3.1. Feature importance methodologies.

To ensure robust feature importance estimates, we implemented two complementary approaches:

1. **Permutation Importance (Ranking-Based):** Model-agnostic method measuring performance decrease when feature values are randomly shuffled [28]. We utilized **Average Precision (AP)** as the scoring metric instead of accuracy or F1-score. This ensures that the importance reflects the feature’s contribution to the model’s *ranking* capability, aligning with our primary research objective.
2. **SHAP (SHapley Additive exPlanations):** Game theory-based approach providing a unified framework for feature attribution [29,30]. This method calculates the marginal contribution of each feature across all possible feature combinations, satisfying efficiency, symmetry, and dummy axioms for theoretically sound importance measures.

**Note on Built-in Importance:** We excluded built-in Gini importance from the final consensus to avoid potential biases toward high-cardinality features [20] and to maintain a focus on model-agnostic interpretability.

**Interpretation Note:** Feature importance reflects associations, not causal effects; prospective validation is required before training interventions.

#### 2.3.2. Consensus methodology.

We computed a consensus feature-importance score by averaging, for each feature, normalized Permutation Importance (AP-based; 10 repeats) and mean absolute SHAP values. To summarize stability, permutation importance was repeated using a fixed random seed, and SHAP values were computed on the full analysis dataset with deterministic LightGBM settings.

### 2.4. Gains chart evaluation framework

#### 2.4.1. Metrics.

We evaluate ranking efficiency with gains charts and lift factors that summarize capture rates at fixed review thresholds (see Introduction for motivation).

**Lift Factor = (% of goal-scoring observations captured in top k%)/ (k%)**

where k is the percentage of top-ranked observations evaluated.

#### 2.4.2. Bootstrap validation protocol.

To quantify uncertainty, we used 1000 bootstrap resamples with replacement; resamples with zero goal-scoring observations were skipped (rare at 5.4% prevalence). For each resample, the model was trained on the bootstrap sample and evaluated on held-out OOB observations to reduce optimistic bias.

**Statistical analysis:**

- Mean capture rates across bootstrap iterations
- 95% percentile confidence intervals [21]
- Lift factors derived as capture rate/ evaluation percentage

To limit inferential inflation, the primary operational metric (Capture Rate @ Top 20%) was pre-specified, and a single permutation test assessed whether ranking performance exceeded chance. Feature-importance stability was summarized via repeated permutation-importance runs and agreement with SHAP rankings.

**Software Implementation:** All analyses were conducted using Python 3.13.5 with the following packages: scikit-learn 1.6.1 for machine learning algorithms and cross-validation, LightGBM 4.6.0 and XGBoost 2.1.1 for gradient boosting, SHAP 0.48.0 for Shapley value calculations, pandas 2.2.3 for data manipulation, numpy 2.1.3 for numerical computations, matplotlib 3.10.0 and seaborn 0.13.2 for figure generation. Statistical analyses and bootstrap validation were implemented with reproducible random seeds (seed = 42) for all stochastic procedures. All package versions were pinned in requirements.txt to ensure reproducible benchmarking and stable library compatibility.

## 3. Results

### 3.1. Algorithm performance comparison

The original dataset contains 2,918 player-match observations from 736 unique players; after excluding 383 goalkeeper observations, 2,535 field player-match observations were analyzed. Goal-scoring prevalence across positions in these observations reflects tactical roles: forwards (12.25%, 81/661), midfielders (5.75%, 55/956), defenders (2.18%, 20/918).

Table 1 summarizes performance across five machine learning algorithms evaluated using nested cross-validation.

**Table 1:** Algorithm performance across models (nested cross-validation).

Algorithm	F1-Score	Capture Rate @ 20% (Mean + /- Std)	Precision	Recall	AUC-PR
LightGBM	0.3084	79.4% + /- 3.1%	0.2406	0.5447	0.3271
Logistic Regression	0.3510	77.2% + /- 4.5%	0.2273	0.7717	0.3162
SVM	0.3383	74.3% + /- 6.5%	0.2612	0.4847	0.2664
Random Forest	0.3236	75.7% + /- 8.4%	0.2102	0.7050	0.2902
XGBoost	0.1983	74.3% + /- 8.1%	0.3992	0.1325	0.2917

**Fig 1:**
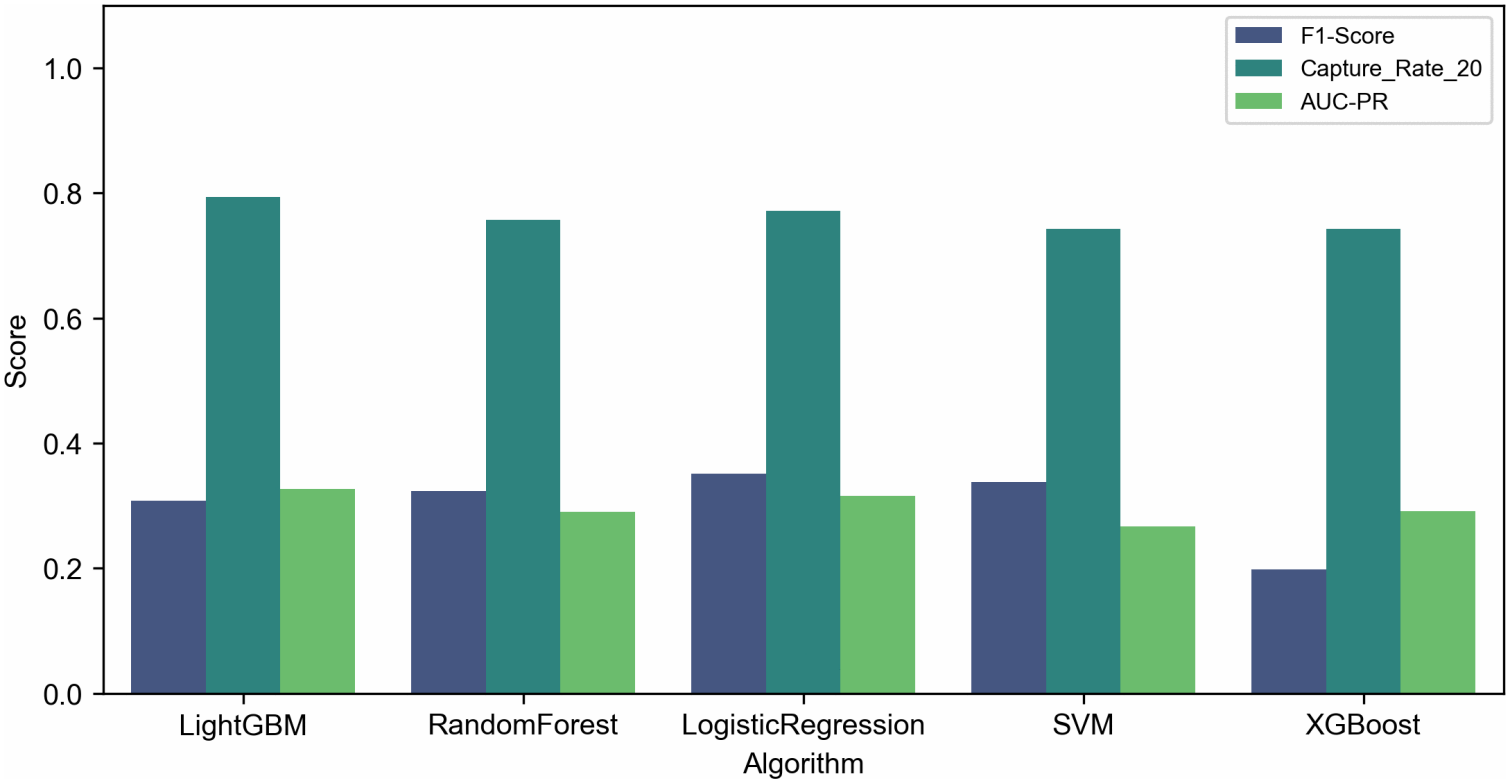
Algorithm performance comparison: classification vs. ranking. The bar chart contrasts F1-scores and Capture Rates at Top 20% across five algorithms (LightGBM, Logistic Regression, SVM, Random Forest, XGBoost).

Fig 1 contrasts F1-scores and Capture Rates at Top 20% across models.

### 3.2. Model selection: ranking-based evaluation priority

We selected the primary model using the pre-specified operational criterion, Capture Rate @ Top 20%, estimated under nested cross-validation (Table 1). LightGBM achieved the highest capture rate at the Top 20% threshold and was therefore used for subsequent ranking-efficiency (Gains Chart) and feature-importance analyses.

### 3.3. Ranking-based efficiency analysis

To quantify ranking efficiency across review budgets and summarize uncertainty, we report a Gains Chart analysis based on an out-of-bag (OOB) bootstrap evaluation.

In this design (n = 1000), the model is trained on each bootstrap sample and evaluated on the held-out OOB observations, reducing optimistic bias compared with evaluating on the same resampled data used for fitting.

Table 2 reports LightGBM capture rates and lift factors across evaluation thresholds.

**Table 2:** Gains chart performance by evaluation threshold (OOB bootstrap, n = 1000).

Threshold	Goal-Scoring Observations Captured	Lift Factor (95% CI)	Efficiency Gain vs. Random
Top 5%	27.1%	5.42 (3.53-7.39)	442%
Top 10%	45.8%	4.58 (3.42-5.64)	358%
Top 15%	61.0%	4.06 (3.33-4.81)	306%
Top 20%	73.9%	3.69 (3.20-4.22)	269%
Top 25%	85.3%	3.41 (3.08-3.73)	241%
Top 30%	94.2%	3.14 (2.89-3.33)	214%

Lift Factor = (% captured/ % evaluated). Confidence intervals are percentile CIs for the capture rate from OOB bootstrap resampling.

Across thresholds, lift factors exceeded 1.0. At the pre-specified 20% review budget, the OOB bootstrap mean capture rate was 73.9% (lift = 3.69x; Table 2). Nested cross-validation results are reported in Table 1.

**Fig 2:**
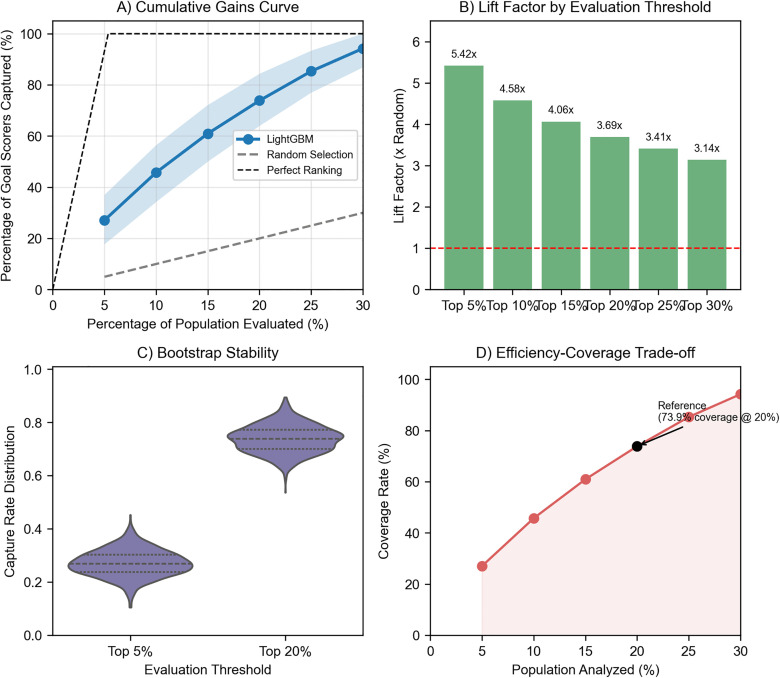
Ranking efficiency and stability across thresholds. **(A)** Cumulative gains curve with 95% OOB bootstrap CI and random selection baseline; **(B)** lift factor by evaluation threshold; **(C)** bootstrap distributions for Top 5% and Top 20% capture rates; **(D)** efficiency-coverage trade-off with the Top 20% threshold highlighted.

Fig 2 presents a multi-panel visualization of the ranking efficiency analysis, consolidating gains curves, lift factors, bootstrap stability, and efficiency-coverage trade-offs.

### 3.4. Feature importance analysis

Permutation and SHAP methods are summarized in the consensus ranking (Table 3).

**Table 3:** Feature importance consensus for goal scoring prediction.

Rank	Feature	Consensus Score	Category
1	AttemptsatGoal	1.000	Expected_Control
2	TotalOffers	0.154	Tactical
3	PassesAttempted	0.090	Technical
4	Ranking202306	0.088	Other
5	JoggingDistance	0.075	Physical
6	TopSpeed	0.060	Physical
7	WalkingDistance	0.057	Physical
8	Defenders	0.051	Other
9	InFront	0.050	Other
10	PushingOn	0.045	Other

**Fig 3:**
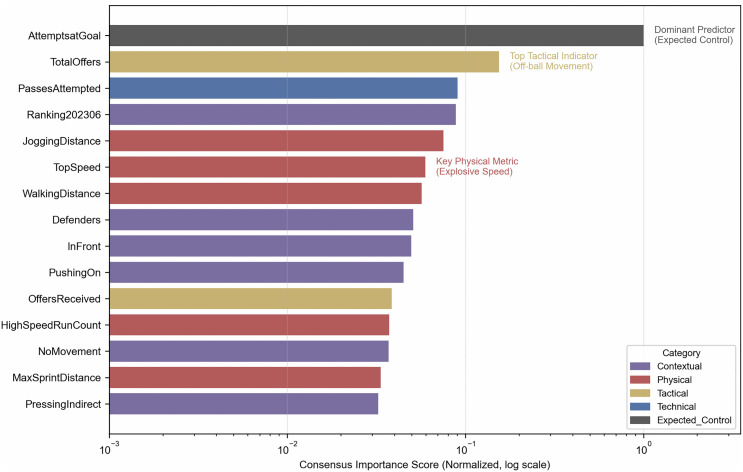
Feature importance consensus: process-oriented metrics vs. controls. The bar chart displays the top 15 features ranked by multi-method consensus score on a log scale. Bars are color-coded by category, and annotations highlight dominant predictors such as Attempts at Goal, Total Offers, and Top Speed.

Table 3 lists features ranked by the consensus score, defined as the average of normalized Permutation Importance and mean absolute SHAP values. In Table 3, *Expected_Control* denotes features that directly capture goal-seeking behavior (e.g., AttemptsatGoal) and are included as a positive control to support model face validity. Fig 3 visualizes this consensus on a log scale with category color coding.

Table 4 aggregates feature importance scores into categories.

**Table 4:** Tactical category importance analysis.

Category	Average Importance	Top Feature	Strategic Interpretation
Expected Control	1.000	AttemptsatGoal	Direct goal-seeking actions
Physical	0.039	JoggingDistance	Sustained workload + speed capacity
Tactical	0.038	TotalOffers	Movement and availability
Technical	0.024	PassesAttempted	Ball-handling and build-up
Other	0.021	Ranking202306	Contextual positioning and team context
Defensive	0.012	Blocks	Ball recovery actions

### 3.5. Position-specific feature importance

SHAP-based importance analysis for each position group identifies the five most influential performance factors for goal scoring within each tactical role (Table 5).

**Table 5:** Position-specific top 5 performance factors for goal scoring (LightGBM).

Position	Rank	Feature	Interpretation
**Forwards** (n = 661)	1	AttemptsatGoal	Direct shooting opportunities
	2	TotalOffers	Active movement to receive ball
	3	PassesAttempted	Link-up play involvement
	4	Ranking202306	Team context signal
	5	TopSpeed	Explosive finishing capacity
**Midfielders** (n = 956)	1	AttemptsatGoal	Direct shooting opportunities
	2	TotalOffers	Active movement to receive ball
	3	Ranking202306	Team context signal
	4	PassesAttempted	Link-up play involvement
	5	JoggingDistance	Sustained work rate
**Defenders** (n = 918)	1	AttemptsatGoal	Direct shooting opportunities
	2	TotalOffers	Active movement to receive ball
	3	Ranking202306	Team context signal
	4	PassesAttempted	Link-up play involvement
	5	WalkingDistance	Recovery and positioning

### 3.6. Operational efficiency and scouting impact

Table 6 reports relative efficiency (lift factors vs. random selection), and Table 7 reports absolute workload metrics (counts of ranked observations and success rates).

**Table 6:** Operational efficiency by evaluation threshold (OOB Bootstrap, n = 1000).

Threshold	Random Selection Capture	Model Selection Capture	Efficiency Improvement
Top 5%	5.0%	27.1%	5.42x
Top 10%	10.0%	45.8%	4.58x
Top 15%	15.0%	61.0%	4.06x
Top 20%	20.0%	73.9%	3.69x
Top 25%	25.0%	85.3%	3.41x
Top 30%	30.0%	94.2%	3.14x

**Table 7:** Scouting efficiency (OOB Bootstrap Mean, n = 1000).

Threshold	Observations Ranked	Goal-Scoring Observations Found	Model Success Rate	Random Success Rate	Fold Improvement
Top 5%	126	36	28.6%	5.4%	5.33x
Top 10%	253	62	24.5%	5.4%	4.57x
Top 15%	380	82	21.6%	5.4%	4.02x
Top 20%	507	100	19.7%	5.4%	3.68x
Top 25%	633	116	18.3%	5.4%	3.42x
Top 30%	760	128	16.8%	5.4%	3.14x

These figures summarize mean OOB results across bootstrap iterations at the observation level. For primary out-of-sample performance estimation, refer to nested CV results in Table 1 (79.4% capture rate at Top 20%). In practice, the ranked observation list is mapped back to players for follow-up review and can be aggregated to a player-level shortlist where identifiers are available.

These figures summarize mean OOB results across bootstrap iterations at the observation level. For primary out-of-sample performance estimation, refer to nested CV results in Table 1 (79.4% capture rate at Top 20%). In practice, the ranked observation list is mapped back to players for follow-up review and can be aggregated to a player-level shortlist where identifiers are available.

## 4. Discussion

### 4.1. Key findings and theoretical implications

**Individual-Focused Talent Identification vs. Team Effects.** We excluded team-dependent variables to emphasize individual player actions over team dominance. LightGBM captures 79.4% of goal-scoring observations at Top 20% in nested CV (Table 1) and 73.9% in OOB bootstrap evaluation (Table 2; lift = 3.69x), indicating that the model concentrates true positives within a limited review budget.

**Ranking-Based Evaluation.** As motivated in the Introduction, ranking-based evaluation aligns with scouting decisions that review only a top fraction of candidates. The gains analysis shows strong enrichment of goal-scoring observations in the top-ranked subset compared with random selection.

**Process-Oriented Feature Importance.** Feature importance highlights tactical availability and build-up involvement (TotalOffers, PassesAttempted) alongside workload indicators (JoggingDistance, TopSpeed). These associations are context-specific and require external and prospective validation before informing training interventions.

### 4.2. Comparison with existing literature

Nested cross-validation reduces selection bias from hyperparameter tuning and improves reproducibility over single splits, aligning with best practices in sports analytics. Relative to prior women’s football ML studies that emphasize classification accuracy, the gains-based evaluation adds an operational lens for resource-limited scouting.

Tournament-format demands may shift the balance between physical and technical indicators relative to seasonal leagues [1,9]; the category-level ratios observed here (Table 4) should be treated as context-specific.

### 4.3. Practical implications

**Scouting and Talent Identification.** Organizations can select review budgets based on Table 7, for example a Top 10% shortlist (253 observations) or a broader Top 20% list (507 observations) that captures most goal-scoring observations in nested CV (Table 1). Observation-level rankings are mapped back to players for follow-up review and can be aggregated to a player-level shortlist when identifiers are available.

**Player Development and Training Design.** Feature importance patterns suggest emphasis on off-ball movement (TotalOffers), build-up involvement (PassesAttempted), and a combination of sustained workload and speed capacity (JoggingDistance, TopSpeed).

### 4.4. Limitations and future research directions

**Study Limitations.** The analysis uses a single tournament context (2023 FIFA Women’s World Cup, N = 2,535) and basic match statistics, which limits generalizability to seasonal play and richer data sources [1,9]. Excluding match-outcome variables reduces threshold-based classification scores relative to outcome-informed models, reflecting the individual-focused design. Prospective validation is required before operational deployment or training interventions.

**Future Research Directions.** Within-sport validation should be prioritized: (1) multi-tournament extension across 2019, 2027 World Cups and UEFA Women’s Champions League to assess threshold stability; (2) seasonal league integration comparing tournament vs. seasonal patterns (NWSL, WSL); (3) enhanced feature integration incorporating xG models and positional tracking data; and (4) prospective validation tracking players identified by the model across 2024–2027 seasons. Cross-domain transferability of gains-based evaluation to other sports remains untested and requires local validation before deployment.

## 5. Conclusions

This study provides a reproducible ranking-based framework for talent identification in women’s football using basic match statistics. Using leakage-free features, nested cross-validation, and bootstrap gains analysis, the model concentrates goal-scoring observations within a limited review budget and highlights consistent performance indicators for scouting. The framework is implementable without tracking data, while thresholds and feature weights should be validated in other competitions.
